# Impact of Satellite Clock Corrections and Different Precise Products on GPS and Galileo Precise Point Positioning Performance

**DOI:** 10.3390/s26020588

**Published:** 2026-01-15

**Authors:** Damian Kiliszek, Karol Korolczuk

**Affiliations:** Faculty of Civil Engineering and Geodesy, Military University of Technology, gen. S. Kaliskiego 2, 00-908 Warsaw, Poland; karol.korolczuk@student.wat.edu.pl

**Keywords:** Precise Point Positioning (PPP), precise products, satellite clock corrections, SP3, CLK, clock interpolation, GPS, Galileo, convergence time, Zenith Total Delay (ZTD)

## Abstract

**Highlights:**

**What are the main findings?**

- Shorter satellite clock sampling (30 s vs. 5 min) yields more accurate PPP and faster convergence time. Final and operational (OPS) products outperform rapid and MGEX; ultra-rapid—especially the predicted half—are the weakest.
- Linear interpolation of clock corrections is constellation-dependent: with 5 min clocks, it benefits Galileo but often harms GPS. In GPS+Galileo, the effects largely cancel, with a slight GPS-driven tendency. With 30 s clocks, interpolation is negligible.

**What is the implication of the main finding?**

- For PPP with 30 s data, use dedicated, dense CLK products from Final/OPS and combine GPS+Galileo; treat ultra-rapid—particularly the predicted segment—cautiously.
- If only 5 min SP3 clocks are available, apply linear interpolation (CLOCK1) for Galileo and nearest-epoch sampling (CLOCK0) for GPS; however, shortening the clock interval matters more than the interpolation strategy.

**Abstract:**

This study assesses how satellite clock products affect Precise Point Positioning (PPP) for GPS, Galileo, and GPS+Galileo. Multi-GNSS data at 30 s were processed for 12 global IGS stations over one week in 2025, with each day split into eight independent three-hour sessions. SP3 clocks (ORB, 5 min) were compared with dedicated CLKs (CLO, 5 s, 30 s, 5 min) across final (FIN), rapid (RAP), and ultra-rapid (ULT; observed/predicted) product lines from multiple analysis centers. Two timing strategies were tested: nearest-epoch sampling (CLOCK0) and linear interpolation (CLOCK1). CLO consistently delivered the lowest 2D/3D errors and the fastest convergence. ORB degraded accuracy by a few millimeters and extended convergence by ~5–10 min, most notably for GPS. With 5 min clocks, CLOCK1 yielded small gains for Galileo but often hurt GPS; with 30 s clocks, interpolation was immaterial; 5 s clocks offered no measurable benefit. FIN outperformed RAP; OPS slightly outperformed MGEX; ESA/GFZ ranked highest. ULT solutions were weaker, especially in the predicted half. Zenith tropospheric delay (ZTD) biases were negligible; variance was smallest for GPS+Galileo with CLO (~7–10 mm), increased by ~1–2 mm with ORB, and was largest in ULT. Dense, high-quality clock products remain essential for reliable PPP.

## 1. Introduction

The technique of absolute positioning, developed over nearly three decades [1,2,3,4,5], has reached a high level of maturity [6]. Within this context, the Precise Point Positioning (PPP) method has become a standard approach across a wide range of scientific and engineering applications [6]. Its versatility is evidenced by numerous uses, including Earth monitoring—e.g., seismology [7], natural hazard monitoring [8], reference frame determination [9], and atmospheric sounding [10,11]—as well as advanced navigation, such as estimating drone trajectories [12], autonomous vehicle trajectories [13], and determining Low Earth Orbit (LEO) satellite orbits [14]. PPP is valued for its high accuracy, achieving millimeter- to centimeter-level precision in static post-processing and decimeter-level precision in kinematic and real-time applications. Moreover, unlike relative positioning methods, PPP does not require connections to reference stations, enabling precise position determination anywhere on Earth and even in its vicinity. This capability is crucial for large-area positioning in remote locations, such as offshore and desert regions, making it ideal for applications like ocean drilling, seafloor mapping, and monitoring geohazards (e.g., volcanic eruptions, earthquakes, and tsunamis) [7]. Nevertheless, its principal limitation, which still warrants further investigation, lies in the long convergence time [6].

In recent years, significant progress has been achieved in Global Navigation Satellite System (GNSS)-based positioning, particularly in the application of the PPP technique. These advancements have resulted from the development of modern GNSS constellations, improvements in mathematical and stochastic modeling, and the increasing availability and precision of GNSS products.

Regarding GNSS constellations, the current era is characterized by the deployment and modernization of multiple global and regional navigation systems. The introduction of new-generation satellites—such as GPS Block III, BeiDou-3 (BDS-3), and the continuous expansion of the Galileo constellation—has significantly improved the overall performance of GNSS. These advancements enhance satellite geometry, signal robustness, and the availability of multi-frequency observations, all of which contribute to improved positioning accuracy and reliability. In parallel, the emergence of high-accuracy augmentation services further strengthens the potential of precise positioning by providing real-time access to orbit and clock corrections. These include the BeiDou PPP-B2b service [15], Japan’s Centimeter-Level Augmentation Service (CLAS) for QZSS [16], and Europe’s Galileo High-Accuracy Service (HAS) [17,18]. While B2b and CLAS are broadcast via geostationary satellites and are thus limited to the Asia–Pacific region, Galileo HAS data are transmitted by a subset of Galileo satellites—currently up to 20—from the overall constellation, enabling global coverage with varying levels of availability [19].

In parallel, methodological advances—most notably the development of PPP with Ambiguity Resolution (PPP-AR) and the routine use of multi-frequency, multi-GNSS observations—have substantially enhanced the reliability and accuracy of PPP [20,21,22]. Integer ambiguity resolution in PPP, as well as in PPP-Real-Time Kinematic (PPP-RTK) [23,24], has been successfully demonstrated in numerous studies and is increasingly being implemented in both post-processed and real-time applications, leading to faster convergence time and more stable positioning solutions [25].

Among the key contributors to the increasing availability and accuracy of GNSS products is the International GNSS Service (IGS), which provides globally accessible, high-quality orbit and clock data essential for PPP [26]. The IGS routinely delivers several categories of precise operational products—final, rapid, and ultra-rapid—that differ in terms of latency and accuracy, as well as real-time (RTS) products supporting operational applications [27,28]. These products are generated by a global network of analysis centers (ACs) and consolidated into official combined solutions coordinated by the IGS Analysis Center Coordinator (ACC) [29].

The Multi-GNSS Experiment (MGEX), initiated by the IGS in 2012, extended the scope of precise orbit and clock determination beyond GPS and GLONASS to include emerging constellations such as Galileo, BeiDou, and QZSS [30,31]. However, the official combined IGS products currently support only GPS and GLONASS, whereas multi-GNSS combined products are still experimental and are distributed as demonstration versions, such as the ultra-rapid product designated ‘IGS0DEMULT’ [32]. These multi-GNSS products are individually generated by several ACs (e.g., COD, GFZ, and WHU), which also provide independent precise orbit and clock solutions. In recent years, a coordinated effort within the IGS community has focused on developing fully integrated multi-GNSS combined products [33,34,35].

A practical implication is that PPP performance varies depending on the AC for different constellations and the specific product line employed [32,36,37,38,39]. Comparative assessments have shown that ACs such as CNES, WHU, GFZ, and CODE often provide among the most consistent and accurate positioning results for both operational (OPS/MGX) and real-time (RTS) products. These differences primarily arise from variations in estimation strategies, network geometry, input data quality, and bias modeling [40].

To achieve reliable PPP solutions, it is essential to simultaneously model (and/or estimate) all significant sources of observation errors and to employ precise satellite orbit and clock products [6,41,42,43,44]. Two main product types are commonly used: SP3 files, which provide precise satellite orbits together with coarser-sampled satellite clock corrections derived from the same orbit–clock adjustment, and CLK files, which deliver dedicated (often denser) satellite—sometimes also station—clock corrections, produced either from the same solution or from a subsequent clock-refinement step, typically at higher temporal resolution [45,46]. Previous studies have shown that, in PPP, the quality of satellite clocks often becomes the dominant factor compared with orbit quality, particularly in kinematic positioning and during the convergence period [47,48,49].

From a practical standpoint, because IGS and AC clock products are published with sampling intervals of 30 s or 5 min, clock values must be interpolated to match the observation epoch during PPP processing [50]. The IGS guidelines recommend linear interpolation, noting that for 5 min sampling the interpolation error for satellite clocks typically remains at the centimeter level [46]. Recent IGS reviews and tutorials summarize current practices and clock modeling approaches, emphasizing that the nominal 5 min resolution necessitates interpolation in PPP, while 30 s products substantially mitigate this effect [51,52]. Experimental studies further demonstrate that short-term clock stability—including high-frequency components—can significantly affect interpolation performance and convergence behavior, especially in kinematic PPP, thereby motivating the use of denser clock products or more advanced interpolation strategies when available [47,48,53].

At the same time, inter-center comparisons have revealed notable differences in the consistency and precision of MGEX and OPS products—on the order of a few centimeters for GPS clocks, and somewhat larger but steadily improving for Galileo [45]. Despite extensive research, there remains a lack of systematic assessment of how satellite clock corrections themselves, as well as their interpolation methods, influence PPP performance within a unified processing framework—particularly for Galileo and combined GPS+Galileo solutions. Earlier studies focused mainly on GPS, comparing different IGS products (ultra-rapid, rapid, and final) and MGEX series, and demonstrated that PPP performance metrics are more sensitive to clock quality than to orbit differences [49]. Those earlier products often provided clock corrections at coarser intervals—sometimes up to 15 min—whereas many current products offer finer sampling, typically 30 s and, in several cases, 5 min.

Most previous studies have typically been conducted for selected time periods using different PPP models, software packages, and precise product sets. As a result, it is not always straightforward to separate product-related effects from differences in processing strategies, or to assess how recent developments in GNSS products translate into PPP performance. In this context, the present work is intended as an application-oriented contribution that provides a uniform benchmark of precise orbit and clock products for conventional float PPP. Accordingly, the aim of this study is to extend this investigation to GPS, Galileo, and combined GPS+Galileo constellations, which represent the most interoperable and mutually compatible GNSS pairing and offer the broadest availability of precise orbit and clock products across multiple families and latency classes. Two temporal strategies for satellite clock handling are considered: (i) linear interpolation of clock corrections and (ii) the use of the nearest available epoch. SP3 products (with jointly estimated orbits and clocks) and CLK products (with dedicated, high-resolution clocks) are compared across final, rapid, and ultra-rapid solutions and multiple product lines (OPS, MGEX, DEM) to quantify the impact of clock characteristics—format, sampling interval, and analysis center—on PPP accuracy, convergence time, and zenith tropospheric delay (ZTD) estimation.

Following the introduction, Section 2 describes the data, product sets, and processing methodology. Section 3 presents the results of the conducted analyses. Section 4 provides a discussion, and Section 5 offers a summary of the key findings.

## 2. Methodology and Data

**Figure 1:**
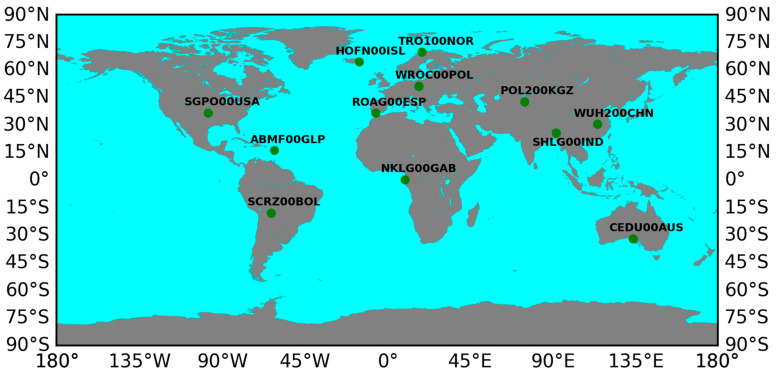
Location of test stations.

This study used observations from 12 globally distributed IGS stations (Figure 1; Table 1), which continuously recorded multi-GNSS data at 30 s intervals over seven days (Day of Year (DoY) 152–158, 2025) and were equipped with a heterogeneous set of receivers and antennas from multiple manufacturers. This period was chosen because it was the most recent interval with complete availability of all products needed for the analysis at the study’s outset. Each day was divided into eight independent three-hour sessions, yielding a total of fifty-six sessions per station across the seven-day dataset for every analyzed solution type and product set. The processing was carried out using the gLAB software package (version 6.0.0), which applies an Extended Kalman Filter (EKF) in conjunction with the conventional PPP model utilizing ionosphere-free float ambiguities [54]. No ambiguity resolution (PPP-AR) or network-assisted PPP-RTK was applied in our processing; all carrier-phase ambiguities were estimated as float values within a conventional ionosphere-free PPP model. This choice reflects the fact that conventional float PPP remains the most widely used PPP approach in many operational and engineering applications. It also allows us to focus the analysis on baseline PPP performance and to isolate the effects of orbit and clock products (and their sampling/interpolation) without the additional benefits introduced by ambiguity fixing or external correction networks.

The undifferenced ionosphere-free linear combinations for GPS and Galileo code and carrier-phase observations (in units of length) are expressed as follows

(1) $${\mathrm{PR}}_{\mathrm{IF},G}=\rho_{r}^{G}+c\left(\delta_{r,\mathrm{clock}}-\delta_{\mathrm{clock}}^{G}\right)+T_{r}^{G}+\varepsilon_{\mathrm{IF}}\;\Phi_{\mathrm{IF},G}=\rho_{r}^{G}+c\left(\delta_{r,\mathrm{clock}}-\delta_{\mathrm{clock}}^{G}\right)+T_{r}^{G}+\lambda_{\mathrm{IF}}^{G}N_{\mathrm{IF}}^{G}+\epsilon_{\mathrm{IF}}\;{\mathrm{PR}}_{\mathrm{IF},E}=\rho_{r}^{E}+c\left(\delta_{r,\mathrm{clock}}-\delta_{\mathrm{clock}}^{E}\right)+{\mathrm{ISB}}_{\mathrm{GE}}+T_{r}^{E}+\varepsilon_{\mathrm{IF}}\;\Phi_{\mathrm{IF},E}=\rho_{r}^{E}+c\left(\delta_{r,\mathrm{clock}}-\delta_{\mathrm{clock}}^{E}\right)+{\mathrm{ISB}}_{\mathrm{GE}}+T_{r}^{E}+\lambda_{\mathrm{IF}}^{E}N_{\mathrm{IF}}^{E}+\epsilon_{\mathrm{IF}}$$

where

- G and E denote the GPS and Galileo systems, respectively;

- ${\mathrm{PR}}_{\mathrm{IF}}$ and $\Phi_{\mathrm{IF}}$ are ionosphere-free linear combination for code and carrier-phase observations, respectively [m];
- $\rho_{r}^{S}$ is the true geometric range between the satellite at the emission time and the receiver at the reception time [m];
- c is the speed of light [m/s];
- $\delta_{r,\mathrm{clock}}$ represent the receiver clock offsets [s];
- $\delta_{\mathrm{clock}}^{S}$ denotes the satellite clock offset for G and E satellites [s];
- ${\mathrm{ISB}}_{\mathrm{GE}}$ is the inter-system bias representing the offset between the time scale and hardware delay of the GPS and Galileo systems [s];
- $T_{r}^{S}$ is the slant tropospheric delay [m];
- $\lambda_{\mathrm{IF}}$ is the wavelength of the ionosphere-free linear combination [m];
- $N_{\mathrm{IF}}$ is the real-valued ambiguity of the ionosphere-free linear combination [cycles];
- $\varepsilon_{\mathrm{IF}}$ and $\epsilon_{\mathrm{IF}}$ are other errors, for example, noise and multipath [m].

In this model, several error sources are considered, such as receiver and satellite antenna phase center offsets, relativistic corrections applied to code and carrier-phase ionosphere-free combinations, carrier-phase wind-up effects, and site displacement corrections following the IERS Conventions [55]. With this setup, the station coordinates, clock correction of the receiver, wet component of the tropospheric delay, and real ambiguity values were estimated. A list of the used products, models, and methods is shown in Table 2 and Table 3.

Several types of precise orbit and clock products provided by the IGS ACs were used in this study, covering final (FIN), rapid (RAP), and ultra-rapid (ULT) solutions. The selected datasets include precise orbit and clock information for both the GPS and Galileo constellations and were obtained from the CDDIS archive (https://cddis.gsfc.nasa.gov/archive/gnss/products/) (accessed on 7 January 2026). Products from COD (CODE AC), ESA (ESA AC), GFZ (GFZ AC), GRG (CNES/CLS AC), WUM/WHU (Wuhan AC), and IGS DEM (the IGS demonstration combination for ultra-rapid) were employed. Processing strategies adopted by each AC are documented in the IGS ACN files [40]. These products are distributed as SP3 orbit files and CLK satellite clock files. SP3 data are provided with a 5 min (05M) interval, spanning one day (01D) for most solutions or two days (02D) for ultra-rapid ones. In turn, CLK products differ in temporal resolution, offering 5 s (05S), 30 s (30S), or 5 min (05M) intervals depending on the analysis center and solution type.

For ultra-rapid products, the two-day files comprise the first 24 h based on actual observations and the subsequent 24 h predicted. These products are updated every 6 h; in this study, we used the 12:00 UTC release, representing solutions with 12 h observed and 12 h predicted. In brief, SP3 files are 01D (FIN/RAP) or 02D (ULT) with a 5 min step; CLK files are provided at 5 s, 30 s, or 5 min (depending on AC and class); and all PPP runs use 30 s observation sampling and 3 h sessions.

To assess the influence of satellite clock interpolation, two approaches were tested:

- CLOCK0, which represents computations without clock interpolation; it selects the clock value from the epoch closest to the observation time;
- CLOCK1, which represents computations with linear interpolation between two adjacent epochs.

For the solutions where 30 s CLK products were available, additional computations were carried out using SP3-based clock corrections—given that the observations were sampled at 30 s intervals—thereby enabling a direct comparison between the CLK and SP3 correction sources. This yielded a set of solutions built on both orbit (SP3) and clock (CLK) inputs provided by different IGS ACs, as summarized in Table 2 and in the later part of this subsection.

Most of the analyzed products followed a consistent structure, with SP3 orbit files at a 5 min interval and CLK products available at a 30 s interval. However, several exceptions were identified within the dataset:

- COD0OPSFIN solutions were available in two variants: one with 30 s CLK products (COD0OPSFIN30s) and another with 5 s CLK products (COD0OPSFIN05s).
- For GFZ0OPSRAP, the clock product was distributed with a 5 min (01D_05M_CLK.CLK) interval, unlike the 30 s standard used in other rapid or final products. This allowed testing both non-interpolated (CLOCK0) and interpolated (CLOCK1) configurations for CLK data with lower temporal resolution.
- The ultra-rapid solutions (COD0OPSULT, GFZ0OPSULT, IGS0DEMULT, and WHU0OPSULT) contained only SP3 orbit products, with no accompanying CLK files. Consequently, PPP computations relied solely on SP3-derived satellite clock corrections, tested in both non-interpolated (CLOCK0_SP3) and linearly interpolated (CLOCK1_SP3) modes. For these ULT products, each daily SP3 series is split into two segments: the first 12 h are observed, and the second 12 h are predicted; results are reported separately for these parts.

These special cases provided an opportunity to evaluate how the temporal resolution and availability of satellite clock products affect PPP performance.

For clarity in discussing results, we use “CLO” to denote solutions that utilize both orbit (SP3) and high-rate clock (CLK) files, and “ORB” for solutions that use only SP3 files (i.e., orbits with their inherent coarse clock corrections).

All PPP processing was conducted using conventional PPP model with a 7° elevation cut-off angle for the GPS, Galileo, and combined GPS+Galileo constellations. Reference coordinates were derived from the daily IGS20 SINEX reference frame, ensuring consistency with the official IGS solutions. The estimated tropospheric delay was subsequently compared with the corresponding official IGS values. Other models and processing methods applied in this study are summarized in Table 3.

## 3. Results

PPP performance was evaluated using three criteria: positioning accuracy, convergence time, and ZTD precision. We report results for two solution families: CLO (SP3 + CLK) and ORB (SP3-only). For each case, we identify the best-performing combination of product class (FIN/RAP/ULT), product line (OPS/MGX/DEM), and analysis center (AC). The influence of clock handling is assessed by comparing CLOCK0 (nearest-epoch) and CLOCK1 (linear interpolation). Ultra-rapid (ULT) products are analyzed separately for the observed and predicted segments.

Performance metrics and criteria

- **Accuracy:** Estimated coordinates were compared with daily IGS20 SINEX reference coordinates and expressed in the NEU frame; 2D and 3D errors were computed accordingly. To reduce the impact of the initial convergence, the first 10 min of each session were excluded only from the accuracy statistics.
- **Convergence time:** Defined using a persistence criterion (continuous satisfaction for ≥20 min):
  ▪ ULT: NEU ≤ 20 cm and 2D/3D ≤ 30 cm.
  ▪ Non-ULT (FIN/RAP): NEU, 2D, and 3D ≤ 10 cm.
    The relaxed thresholds for ULT reflect that achieving 10 cm within 3 h sessions—especially in the predicted half—is rarely feasible, whereas the 30 cm criterion enables an informative comparison and still represents decimeter-level performance relevant to real/near-real-time applications.
- **ZTD:** ZTD consistency is evaluated against the IGS reference values (IGS0OPSFIN).

### 3.1. Analysis of Positioning Accuracy and Convergence Time

**Figure 2:**
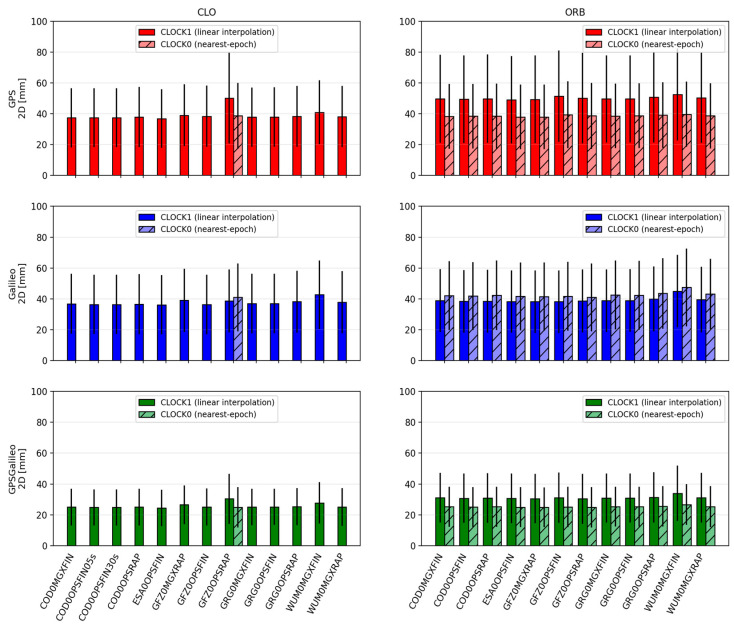
Mean ± standard deviation of 2D position errors for CLO (**left**) and ORB (**right**) solutions across IGS ACs. Darker bars denote CLOCK1 (linear interpolation), lighter bars denote CLOCK0 (nearest-epoch).

**Figure 3:**
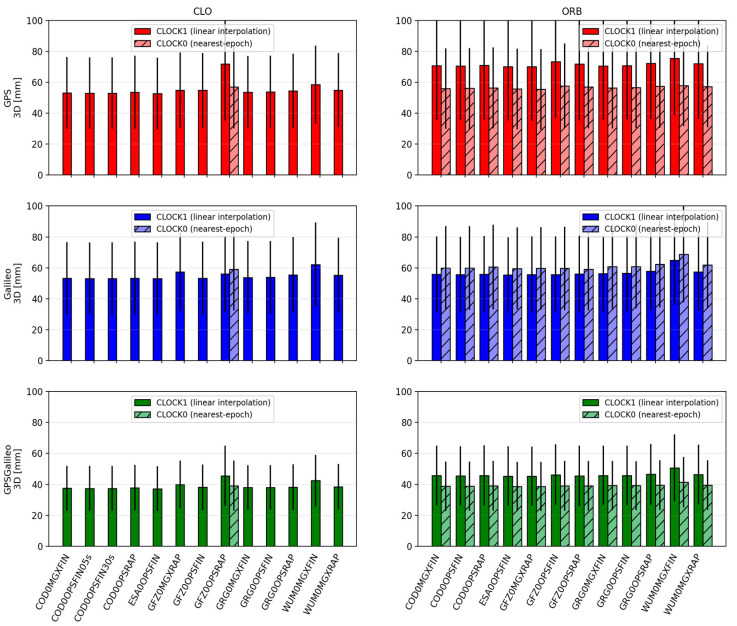
Mean ± standard deviation of 3D position errors for CLO (**left**) and ORB (**right**) solutions across IGS ACs. Darker bars denote CLOCK1 (linear interpolation), lighter bars denote CLOCK0 (nearest-epoch).

**Figure 4:**
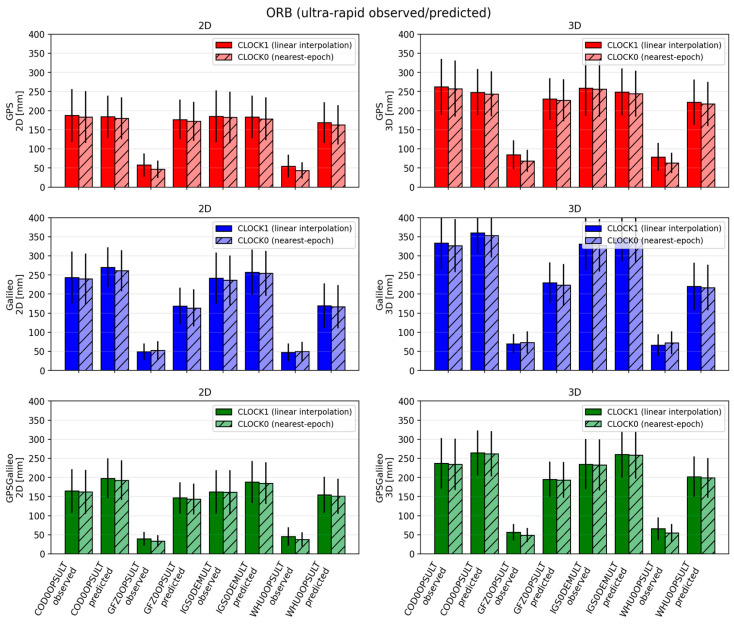
Mean ± standard deviation of 2D (**left**) and 3D (**right**) position errors for ORB solutions across IGS Acs for ultra-rapid products. Darker bars denote CLOCK1 (linear interpolation), lighter bars denote CLOCK0 (nearest-epoch).

**Figure 5:**
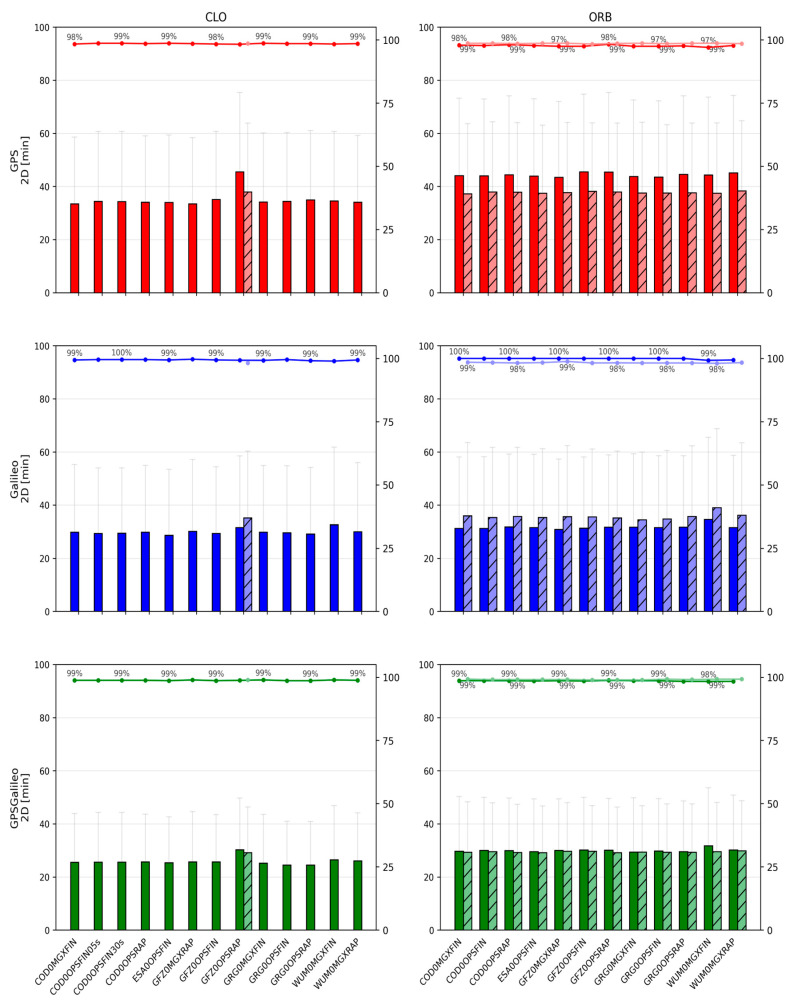
Mean convergence time ± standard deviation of 2D for CLO (**left**) and ORB (**right**) solutions across IGS ACs. Darker bars denote CLOCK1 (linear interpolation), lighter bars denote CLOCK0 (nearest-epoch). The percentages shown above the bars indicate the fraction of sessions that achieved convergence time under the adopted persistence criteria.

**Figure 6:**
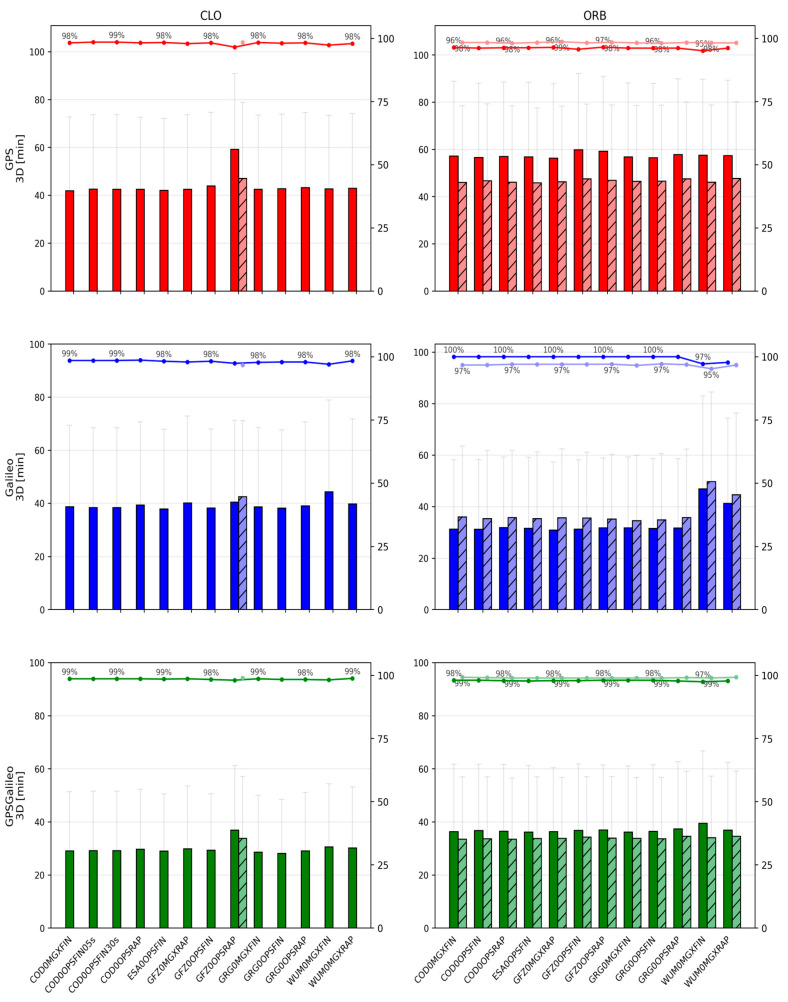
Mean convergence time ± standard deviation of 3D CLO (**left**) and ORB (**right**) solutions across IGS ACs. Darker bars denote CLOCK1 (linear interpolation), lighter bars denote CLOCK0 (nearest-epoch). The percentages shown above the bars indicate the fraction of sessions that achieved convergence time under the adopted persistence criteria.

**Figure 7:**
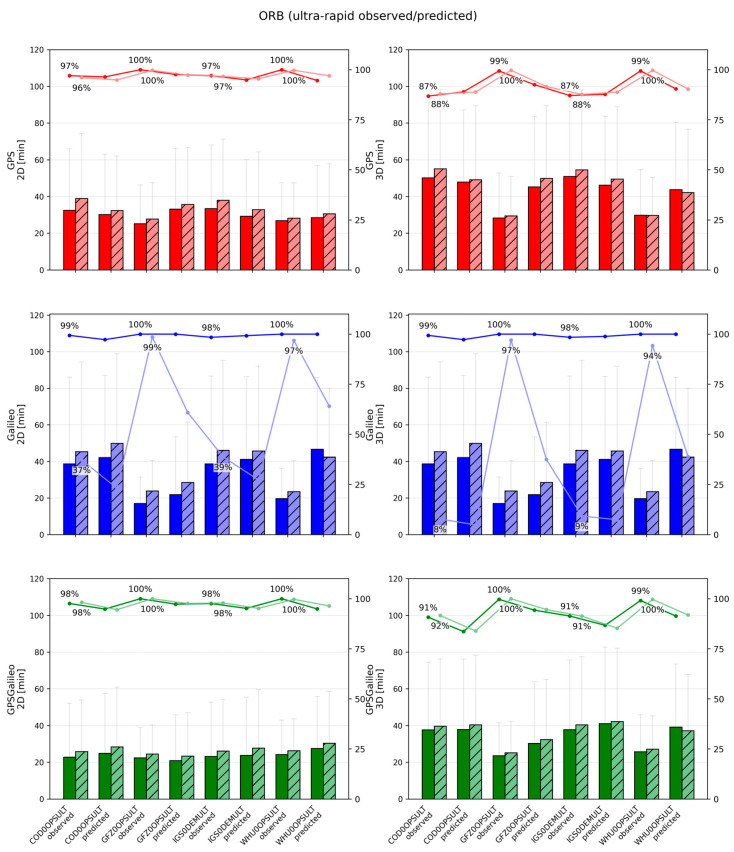
Mean convergence time ± standard deviation of 2D (**left**) and 3D (**right**) for ORB solutions across IGS Acs for ultra-rapid products. Darker bars denote CLOCK1 (linear interpolation), lighter bars denote CLOCK0 (nearest-epoch). The percentages shown above the bars indicate the fraction of sessions that achieved convergence time under the adopted persistence criteria.

Figure 2, Figure 3, Figure 4, Figure 5, Figure 6 and Figure 7 summarize positioning accuracy and convergence performance. Satellite availability was monitored and remained essentially constant across scenarios (mean ≈8.8 satellites for GPS, ≈8.0 for Galileo, and ≈16.8 for GPS+Galileo). The only exception was the WHU ultra-rapid case, where GPS+Galileo dropped to ≈12 satellites; this did not materially affect the conclusions and is not further analyzed.

#### 3.1.1. CLO Solutions Using CLK and SP3 Products

The CLO solutions (based on SP3 orbits combined with dedicated CLK files) provide the best overall PPP performance. Across all constellations, the achieved accuracy is at the centimeter level, and the convergence behavior is stable. The GPS+Galileo combination consistently outperforms single-constellation solutions, both in terms of accuracy and convergence time, which reflects the improved satellite geometry and redundancy in the combined case.

For GPS+Galileo, the mean 3D error lies in a relatively narrow range of about 37.2–42.4 mm, with 2D errors of about 24.4–27.8 mm. The best performance within this group is obtained with ESA0OPSFIN (≈37.2 mm 3D; ≈24.4 mm 2D), while the weakest is WUM0MGXFIN (≈42.4 mm 3D; ≈27.8 mm 2D). Importantly, even the “weaker” solutions remain close to the best-case performance, indicating that in CLO mode the differences among high-quality product streams are limited to a few millimeters.

In the single-constellation cases, the errors are larger and the spread increases. For GPS-only PPP, the mean 3D error is approximately 52.6–64.4 mm (2D: 36.9–44.4 mm). The lowest errors again occur for ESA0OPSFIN, while the upper end of the range is represented by GFZ0OPSRAP. For Galileo-only PPP, the mean 3D error is approximately 53.0–62.2 mm (2D: 36.2–42.7 mm), with ESA0OPSFIN also providing the most accurate results, and WUM0MGXFIN representing the upper end. These outcomes reinforce that the combined GPS+Galileo configuration is consistently the most robust, while single-constellation solutions remain more sensitive to product differences.

Convergence statistics in CLO mode are similarly stable. In nearly all cases, convergence success is close to 100% (typically 98–100% across sessions). The GPS+Galileo solutions converge the fastest, with mean 3D convergence times commonly around 22–26 min, whereas single-constellation solutions require longer times: roughly 28–34 min for GPS and 27–33 min for Galileo. The only clearly distinct behavior within CLO is observed for GFZ0OPSRAP, which uses a 5 min clock interval and consequently shows slower and less uniform convergence (typically by ~5–10 min compared to solutions that use 30 s CLKs).

Two comparisons illustrate the role of clock sampling density. First, for COD0OPSFIN, CLK products are available at 30 s and 5 s intervals. When processed with 30 s observations, the higher clock rate does not translate into measurable gains: 3D errors remain essentially identical (e.g., GPS: 53.0 mm for both; Galileo: 53.1 mm for both; GPS+Galileo: 37.4 mm for both), and convergence times do not change in a meaningful way. Second, when the clock interval becomes coarse (as in the GFZ0OPSRAP 5 min clock case), both accuracy and convergence degrade noticeably, confirming that for conventional PPP with 30 s data, using dense and high-quality clock products is more important than pushing clock sampling below the observation interval.

Finally, within the CLO family, a small but repeatable advantage is visible for FIN vs. RAP and for OPS vs. MGX, typically at the level of a few millimeters in 2D/3D errors. While these differences are modest, they are consistent across constellations. The markedly different behavior appears only when moving from FIN/RAP to ultra-rapid products, which is treated separately below.

#### 3.1.2. ORB Solutions Using Only SP3 Products

In the ORB solutions, the PPP processing relies solely on SP3 products, meaning that the satellite clock corrections are taken from the SP3 files themselves and are sampled at a 5 min interval. Compared to CLO, where dedicated CLKs are often available at 30 s sampling, ORB solutions represent a “sparse clock” scenario. This difference is reflected in both accuracy and convergence, especially for single-constellation PPP.

For GPS+Galileo, the ORB results remain relatively close to those obtained in CLO mode. Mean 3D errors are typically around 38–43 mm (2D: 25–29 mm), i.e., only a few millimeters worse than CLO. The best results occur for solutions such as GFZ0MGXRAP and ESA0OPSFIN (e.g., ~38.6 mm 3D for GFZ0MGXRAP). Although some MGX/RAP combinations show larger scatter, the overall message is that in the combined GPS+Galileo case, the geometry and redundancy partially compensate for the coarse clock sampling.

The single-constellation ORB solutions show a clearer deterioration relative to CLO. For GPS-only, mean 3D errors increase to approximately 55–66 mm (2D: 38–45 mm), while for Galileo-only, mean 3D errors are roughly 55–62 mm (2D: 36–43 mm). The degradation is generally stronger for GPS than for Galileo, consistent with the higher sensitivity of GPS-only PPP to the sparse 5 min clock step in the SP3 products.

Convergence behavior in ORB mode remains stable in terms of success rate—typically 96–100% of sessions converge—but convergence times are consistently longer than for CLO. For GPS+Galileo, mean 3D convergence times are often about 27–32 min, which is approximately 5–7 min slower than the corresponding CLO solutions. Single-constellation solutions show a stronger penalty: GPS often requires ~34–40 min, and Galileo ~32–38 min, reflecting the greater sensitivity of single-system PPP to sparse clocks. As in CLO, FIN tends to outperform RAP, and OPS tends to slightly outperform MGX, but the dominant factor in ORB mode is the 5 min clock sampling, which makes ORB systematically worse than CLO even when the orbit quality is high.

#### 3.1.3. Effect of Clock Interpolation (CLOCK1) vs. Nearest-Epoch (CLOCK0)

The influence of clock handling is meaningful primarily in scenarios where clock values are sparse, i.e., when clocks are available only at 5 min sampling. This is why CLOCK0 vs. CLOCK1 was evaluated mainly for ORB solutions (SP3 clocks) and for the one CLO case where CLK sampling is also 5 min (GFZ0OPSRAP).

A consistent pattern emerges: the impact of interpolation is constellation-dependent. For Galileo, linear interpolation (CLOCK1) tends to slightly improve performance. The improvements are small but repeatable: 2D and 3D errors decrease by a few millimeters, and convergence times shorten by a few minutes relative to using the nearest-epoch (CLOCK0). In contrast, for GPS, CLOCK1 often degrades the results: the 2D/3D errors increase by several millimeters, and convergence tends to be slower. For the combined GPS+Galileo solution, these opposite trends largely cancel out, so the differences between CLOCK0 and CLOCK1 are usually marginal—often just a few millimeters in error and a minute or two in convergence time, with a slight tendency toward CLOCK0 being better due to the GPS behavior.

Some dependence on analysis center can be observed, but it is clearly secondary compared with the constellation effect. For example, the Galileo gains from interpolation appear more consistently in certain product streams (e.g., GFZ/ESA), while GPS degradation under CLOCK1 is more visible in others (e.g., WUM/GRG). Nevertheless, the general conclusion remains robust across products: CLOCK1 is typically beneficial for Galileo when clocks are sparse, whereas CLOCK0 is typically safer for GPS. This also aligns with the broader interpretation that interpolation can help when clock noise at short time scales is low, but it can propagate noise or local inconsistencies when short-term noise is larger.

For the CLO case with 5 min CLKs (GFZ0OPSRAP), interpolation yields a modest gain, improving accuracy by a few millimeters and convergence by a few minutes. However, even in this “best” 5 min CLK case, the performance remains distinctly worse than CLO solutions using 30 s clocks. This comparison confirms that clock sampling density dominates: the choice between CLOCK0 and CLOCK1 matters under 5 min sampling, but it is still much less important than switching from 5 min to 30 s clock updates.

#### 3.1.4. Performance of Ultra-Rapid (ULT) Products (Observed vs. Predicted)

Ultra-rapid products represent the most challenging scenario in this study because they provide only SP3 products (with 5 min clocks) and are designed for near-real-time use. Moreover, each ultra-rapid product contains two segments: an observed part (first 12 h) and a predicted part (next 12 h). Because the predicted part contains forecasted orbits/clocks, its quality is generally lower, which directly affects PPP.

In terms of accuracy, the ULT solutions are clearly worse than the FIN/RAP solutions. For GPS, 2D errors span roughly 50–190 mm and 3D errors 70–260 mm. GFZ0OPSULT and WHU0OPSULT provide the most stable and accurate behavior, with relatively small differences between observed and predicted segments (often around 50–60 mm 2D and 70–90 mm 3D). In contrast, COD0OPSULT and the demonstration combination IGS0DEMULT show substantially larger errors (roughly 180–190 mm 2D and 230–260 mm 3D).

For Galileo, the spread is wider: 2D errors range approximately 45–270 mm, and 3D errors 70–360 mm. The same general ranking holds—GFZ and WHU are best—while COD and IGS0DEM are weakest, especially in the predicted segment. For the combined GPS+Galileo case, typical values cluster around 140–200 mm (2D) and 200–270 mm (3D), again with GFZ/WHU being the most consistent, and COD/IGS0DEM exhibiting larger errors and stronger degradation in the predicted part.

Convergence performance in ULT is also more variable, which motivated the use of relaxed convergence thresholds for ULT (decimeter-level). GPS convergence times typically fall around 25–40 min (2D) and 30–55 min (3D), with longer and less stable convergence in the predicted segment and for the weaker product streams. Galileo convergence can be fast in good cases but can also fail to meet the threshold in the predicted part for weaker products; in those cases, convergence success can drop sharply. The combined GPS+Galileo solution is generally more reliable, with convergence around 22–30 min (2D) and 30–45 min (3D), and success rates typically 90–100%, particularly for GFZ and WHU.

CLOCK handling has a smaller role than product quality in ULT, but it is still visible. Linear interpolation (CLOCK1) provides a modest improvement, commonly reducing errors by a few millimeters (sometimes up to ~10 mm) and improving convergence by roughly 2–5 min, especially for Galileo and in the predicted segment. However, the dominant factor remains the quality of the ultra-rapid product itself—particularly whether the solution relies on a stronger AC stream (GFZ/WHU) or a weaker one (COD/IGS0DEM). Overall, the ULT analysis confirms that while interpolation can help under sparse clock sampling, it cannot compensate for the fundamental limitations of 5 min clocks and predicted corrections.

### 3.2. Analysis of ZTD

**Figure 8:**
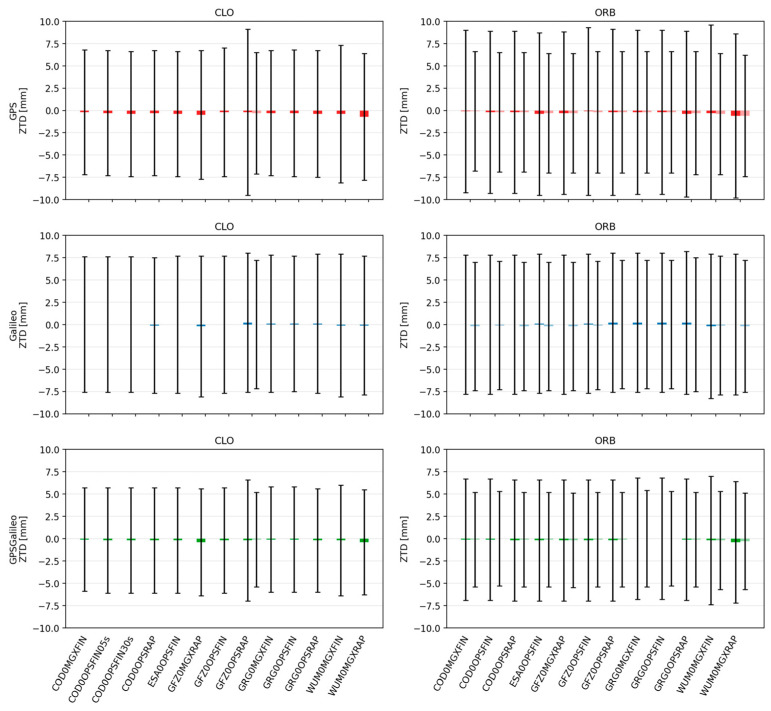
Mean ± standard deviation of ZTD for CLO (**left**) and ORB (**right**) solutions across IGS AC products. Darker bars denote CLOCK1 (linear interpolation), lighter bars denote CLOCK0 (nearest-epoch).

**Figure 9:**
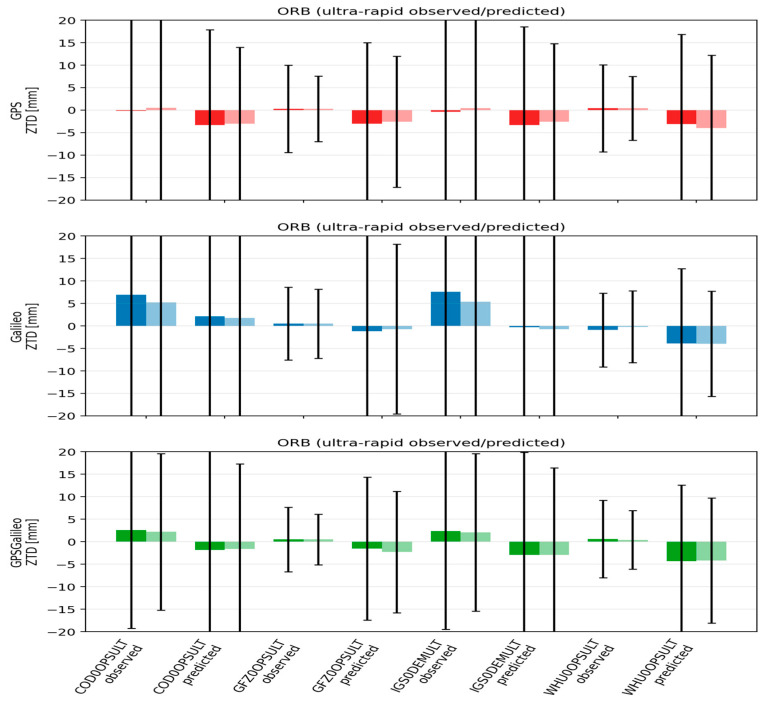
Mean ± standard deviation of ZTD for ORB solutions across IGS AC for ultra-rapid products. Darker bars denote CLOCK1 (linear interpolation), lighter bars denote CLOCK0 (nearest-epoch).

Beyond coordinate accuracy, PPP also provides estimates of tropospheric parameters. We evaluated ZTD precision by comparing PPP-estimated values against the IGS reference troposphere product (IGS0OPSFIN). We report both bias (mean difference) and scatter (standard deviation), because in most cases the mean bias is small and the variance becomes the key indicator of product quality. From a practical perspective, GNSS meteorology and NWP assimilation often cite a target level around 0.6 cm and an upper limit around 3 cm for real-time ZTD applications [11]. Against this background, we discuss how product class (FIN/RAP/ULT), clock source (CLO vs. ORB), and interpolation strategy affect ZTD behavior. The results of this analysis are presented in Figure 8 and Figure 9.

For FIN/RAP solutions, ZTD biases are generally negligible, with means typically within about ±1–2 mm. The main differences appear in the scatter. The most stable ZTD estimates are obtained in CLO mode, particularly in the GPS+Galileo configuration, where the standard deviation is typically ~7–10 mm. Single-constellation solutions are usually slightly noisier by about 1–2 mm, which is consistent with the reduced observation redundancy and weaker geometry.

Comparing FIN and RAP, FIN tends to be marginally more stable, often by roughly 0.5–1.5 mm in standard deviation, while OPS may be slightly tighter than MGX. Differences among analysis centers are smaller than in the positioning metrics, but some AC streams (e.g., GFZ/WUM) tend to provide slightly tighter ZTD distributions than others (e.g., COD/GRG), depending on the constellation and product line.

The ZTD behavior becomes clearly worse in the ULT class. Mean biases remain small, but the scatter increases substantially, typically to around 10–18 mm, with the predicted segment being noisier than the observed segment by roughly 1–3 mm. AC differences are also more pronounced under ULT: stronger streams such as GFZ (and in some cases WUM) yield noticeably tighter ZTD distributions, while COD and especially IGS0DEM can be noisier, particularly in the predicted half. CLOCK1 can slightly reduce scatter in ULT, but again the improvement is small compared to product class and AC effects.

Overall, the ZTD results mirror the coordinate results: biases are generally negligible, and ZTD precision degrades from CLO to ORB and then to ULT, with the predicted ultra-rapid segment being the most variable. At the same time, even the ULT ZTD scatter remains well within typical NWP real-time thresholds, and the best configurations approach the commonly cited target accuracy. Combining GPS and Galileo consistently improves ZTD stability, reinforcing the practical advantage of multi-GNSS PPP not only for positioning but also for atmospheric parameter estimation.

## 4. Discussion

The results clearly show that satellite clock characteristics—format, update interval, and source (AC)—are among the key factors limiting PPP performance, in terms of accuracy, convergence time, and ZTD stability. Within the CLO family (SP3+CLK products), where dense clock corrections are available (typically 30 s), the resulting 2D/3D errors are the lowest and convergence times the shortest. In ORB mode (SP3-only products, 5 min), a systematic increase in errors (by several millimeters) and longer convergence time (~5–10 min) are observed, particularly for single-constellation GPS. This is consistent with reports in the literature emphasizing the benefits of dense clock corrections and higher-grade IGS products (final/rapid over ultra-rapid) in PPP [49].

Combining constellations (GPS+Galileo) consistently improves solution stability and shortens convergence time relative to single-constellation PPP. In our analysis, this is reflected by tightly clustered error distributions for GPS+Galileo (approximately 24–28 mm 2D and 37–42 mm 3D in the best CLO cases) and convergence rates near 100%, whereas GPS-only or Galileo-only solutions have broader error ranges and slightly lower convergence success.

The clock correction interval and strategy lead to two practical conclusions. First, when dense CLK corrections (30 s) are available, there is no measurable gain from even denser clocks (e.g., COD 5 s) at a 30 s observation interval—neither accuracy nor convergence time improves. In contrast, when only coarse 5 min updates (SP3) are available, CLO clearly outperforms ORB, and performance degrades in families where the only clocks have a 5 min step (e.g., GFZ0OPSRAP). Second, linear interpolation (CLOCK1) applied to 5 min series has a mixed, constellation-dependent impact: for Galileo, it brings a small but repeatable benefit (lower 2D/3D errors and convergence time faster by a few minutes), whereas for GPS it can be unfavorable (higher errors and longer convergence time). In the combined GPS+Galileo mode, these effects partially cancel, and differences relative to CLOCK0 are generally marginal. This suggests that under sparse 5 min sampling, interpolation may smooth short-period noise where clocks exhibit higher short-term stability (Galileo), but can introduce modeling error in the presence of larger noise (certain GPS series/satellites) [52,56].

The observed superior response of Galileo to interpolation over short horizons (≈30–300 s) is consistent with GNSS clock stability analyses: the PHM+Rb ensemble on Galileo satellites is among the most short-term stable (e.g., ADEV ≈ 1.2 × 10^−13^ at 30 s and ≈1.7 × 10^−14^ at 900 s), which reduces noise in clock products and limits interpolation-induced degradation. Over longer horizons, differences relative to the newer GPS generations (IIF/III with improved Rb) diminish, and the resulting performance is often comparable [56].

Across the CLO and ORB families, there is a small but repeatable advantage of FIN over RAP and OPS over MGX (gains of a few millimeters and a few minutes of convergence time). These conclusions are consistent with earlier PPP studies, which confirm the benefits of denser clock corrections and higher-class IGS products over ULT solutions [49]. Differences between ACs are smaller than the impact of the clock update interval; nevertheless, the most stable series (e.g., ESA/GFZ/WHU) consistently rank among the best, whereas configurations with 5 min clocks (e.g., GFZ0OPSRAP) lag behind. The observed inter-AC performance differences can likely be attributed to each AC’s specific processing strategies and constraints in their orbit/clock determination [40].

In ULT mode (a near-real-time scenario where only 5 min SP3 clocks are available), the results are clearly weaker than in the CLO/ORB final or rapid solutions. The predicted segment is often substantially worse than the observed one: GFZ/WHU maintain high convergence rates and lower errors, while COD/IGS0DEM exhibit greater variability, especially for Galileo in the predicted portion. Interpolation (CLOCK1) in ULT yields a moderate improvement (several to ~10 mm and 2–5 min) but does not compensate for the limitations of 5 min sampling.

With respect to ZTD, biases are negligible across all configurations; quality is governed by the variance, which is smallest in CLO (GPS+Galileo ~7–10 mm), slightly larger in ORB (+1–2 mm), and largest in ULT (typically ~10–18 mm; with the predicted segment usually worse than observed). The influence of interpolation is smaller than the choice of product class/line and AC, as well as the benefit from constellation combination. This ordering of ZTD stability and sensitivity to product class is consistent with previous findings across IGS/MGEX lines.

In summary, the dominant factor for PPP performance is the density and quality of the satellite clock corrections (30 s ≫ 5 min). Next in importance are the product time category (FIN/RAP > ULT), then the product line (OPS > MGX), followed by the choice of AC. The interpolation strategy for satellite clock corrections (CLOCK0/CLOCK1) becomes relevant under sparse (5 min) sampling and is constellation-dependent: generally beneficial for Galileo but requiring caution for GPS; in combined GPS+Galileo, the net effect is usually small. These conclusions align with prior PPP studies and GNSS clock stability analyses, including the higher short-term stability of Galileo clocks (PHM+Rb) [49,56].

## 5. Conclusions

Analyses confirm that the density and quality of satellite clock corrections are the primary determinants of PPP performance. CLK-based corrections at a 30 s interval (CLO solutions) consistently yield the smallest 2D/3D position errors and the shortest convergence times. In contrast, using clock corrections derived from a 5 min SP3 interval (ORB or ULT cases) increases position errors by a few millimeters and typically prolongs convergence by ~5–10 min—most markedly in single-constellation GPS solutions. Sensitivity to the clock update interval is constellation-dependent: GPS accuracy and convergence degrade more under sparse 5 min sampling than Galileo, while combining GPS+Galileo mitigates some of the degradation through partial error cancelation. With 5 min clocks, linear interpolation (CLOCK1) usually provides a small but repeatable gain for Galileo (improving errors by a few millimeters and convergence by a few minutes) and can be unfavorable for GPS; in GPS+Galileo, differences between CLOCK1 and CLOCK0 are generally minor. When dense 30 s CLK products are available, the choice between CLOCK1 and CLOCK0 becomes almost irrelevant compared to the large benefit of the dense sampling itself.

Across analysis centers, the best and most stable performance was obtained using final or operational products from top-performing ACs (e.g., ESA, GFZ), whereas configurations based on 5 min clocks (e.g., GFZ0OPSRAP) consistently lagged. Within the ORB family, differences among ACs were smaller than the impact of the 5 min update interval, although ESA/GFZ solutions remained among the top performers. In the ultra-rapid class—where only 5 min SP3 clocks are available—PPP results were substantially weaker than in final/rapid cases, with the predicted half of the product performing significantly worse than the observed half. Even in these ULT cases, GFZ and WHU products showed relatively better stability, whereas COD and IGS0DEM were more erratic, particularly for Galileo during prediction intervals. Applying linear interpolation in the ultra-rapid solutions provided a slight improvement but did not overcome the inherent limitations of the sparse clock data and prediction errors.

For ZTD estimation, all solutions showed negligible bias relative to the IGS references, and the precision of ZTD followed the product hierarchy: highest in CLO (~7–10 mm scatter for GPS+Galileo), slightly worse in ORB (+1–2 mm), and lowest in ULT (~10–18 mm, with observed segments better than predicted). GPS+Galileo combinations consistently yielded more stable ZTD estimates than single-constellation cases, demonstrating the value of multi-GNSS data for tropospheric retrieval. The effect of clock interpolation on ZTD scatter was marginal and observable mainly in the 5 min clock scenarios.

Practical recommendations: For static PPP with 30 s observation sampling, the use of CLK products with 30 s satellite clocks (e.g., IGS Final or equivalent operational products) is recommended, as these produce the best accuracy and the fastest convergence time. When only 5 min SP3 clock products are available, it is advisable to apply linear interpolation (CLOCK1) for Galileo but use nearest-epoch sampling (CLOCK0) for GPS; the results showed that interpolation can slightly improve Galileo’s solution but may degrade GPS. In combined GPS+Galileo PPP, the choice of interpolation strategy is less critical because the constellation-specific interpolation effects tend to offset each other; in such cases, it is more important to select high-quality products (final over rapid, OPS over MGEX) from reliable ACs to achieve optimal performance.

It should be noted that this study was limited to a one week static PPP analysis focusing on GPS and Galileo only. Including additional constellations (e.g., BeiDou, QZSS) would likely further improve PPP performance by increasing satellite availability and strengthening geometry; however, this was beyond the scope of the present study. Moreover, because only static sessions were analyzed, the reported results likely represent an optimistic scenario. In kinematic PPP (e.g., a moving receiver or real-time applications), the effects of sparse clock updates and interpolation errors may be more pronounced due to reduced averaging and receiver dynamics. Future work should therefore consider kinematic PPP and full multi-constellation processing to further enhance PPP performance and to verify whether the strategies identified here remain valid under more dynamic, multi-GNSS conditions.

## Figures and Tables

**Table 1:** Equipment used in the IGS stations.

Station	Receiver	Antenna
SGPO00USA	JAVAD TRE_3S	JAVRINGANT_G5T
POL200KGZ	JAVAD TRE_3 DELTA	TPSCR.G3
WUH200CHN	JAVAD TRE_3	JAVRINGANT_G5T
WROC00POL	LEICA GR30	LEIAR25.R4
SCRZ00BOL	LEICA GR10	LEIAR10
HOFN00ISL	LEICA GR50	LEIAR25.R4
ABMF00GLP	SEPT POLARX5	TRM57971.00
NKLG00GAB	SEPT POLARX5	TRM59800.00
ROAG00ESP	SEPT POLARX5TR	LEIAR25.R4
SHLG00IND	TRIMBLE ALLOY	LEIAR25.R3
TRO100NOR	TRIMBLE NETR9	TRM59800.00
CEDU00AUS	TRIMBLE ALLOY	TWIVC6050

**Table 2:** Products used in the analysis, with native intervals, solution names, and clock interpolation settings. CLO = PPP using CLK files; ORB = PPP using SP3-derived clock corrections. CLOCK0 = nearest-epoch (no interpolation); CLOCK1 = linear interpolation. NONE = configuration not run / not applicable—either because CLK files are unavailable (e.g., ultra-rapid products) or because, for CLK = 30 s with 30 s observations, CLOCK0 ≡ CLOCK1 (only CLOCK1 reported). Exception: GFZ0OPSRAP has CLK = 5 min, so both CLOCK0 and CLOCK1 were evaluated.

Solution/Products	Products with Interval		Types of Solutions for Analysis			
	CLK	SP3	CLO		ORB	
COD0MGXFIN	01D_30S_CLK.CLK	01D_05M_ORB.SP3	NONE	CLOCK1_CLO	CLOCK0_ORB	CLOCK1_ORB
COD0OPSFIN05s	01D_05S_CLK.CLK	01D_05M_ORB.SP3	NONE	CLOCK1_CLO	NONE	
COD0OPSFIN30s	01D_30S_CLK.CLK	01D_05M_ORB.SP3	NONE	CLOCK1_CLO	CLOCK0_ORB	CLOCK1_ORB
COD0OPSRAP	01D_30S_CLK.CLK	01D_05M_ORB.SP3	NONE	CLOCK1_CLO	CLOCK0_ORB	CLOCK1_ORB
ESA0OPSFIN	01D_30S_CLK.CLK	01D_05M_ORB.SP3	NONE	CLOCK1_CLO	CLOCK0_ORB	CLOCK1_ORB
GFZ0MGXRAP	01D_30S_CLK.CLK	01D_05M_ORB.SP3	NONE	CLOCK1_CLO	CLOCK0_ORB	CLOCK1_ORB
GFZ0OPSFIN	01D_30S_CLK.CLK	01D_05M_ORB.SP3	NONE	CLOCK1_CLO	CLOCK0_ORB	CLOCK1_ORB
GFZ0OPSRAP	01D_05M_CLK.CLK	01D_05M_ORB.SP3	CLOCK0_CLO	CLOCK1_CLO	CLOCK0_ORB	CLOCK1_ORB
GRG0MGXFIN	01D_30S_CLK.CLK	01D_05M_ORB.SP3	NONE	CLOCK1_CLO	CLOCK0_ORB	CLOCK1_ORB
GRG0OPSFIN	01D_30S_CLK.CLK	01D_05M_ORB.SP3	NONE	CLOCK1_CLO	CLOCK0_ORB	CLOCK1_ORB
GRG0OPSRAP	01D_30S_CLK.CLK	01D_05M_ORB.SP3	NONE	CLOCK1_CLO	CLOCK0_ORB	CLOCK1_ORB
WUM0MGXFIN	01D_30S_CLK.CLK	01D_05M_ORB.SP3	NONE	CLOCK1_CLO	CLOCK0_ORB	CLOCK1_ORB
WUM0MGXRAP	01D_30S_CLK.CLK	01D_05M_ORB.SP3	NONE	CLOCK1_CLO	CLOCK0_ORB	CLOCK1_ORB
COD0OPSULT	NONE	02D_05M_ORB.SP3	NONE		CLOCK0_ORB	CLOCK1_ORB
GFZ0OPSULT	NONE	02D_05M_ORB.SP3	NONE		CLOCK0_ORB	CLOCK1_ORB
IGS0DEMULT	NONE	02D_05M_ORB.SP3	NONE		CLOCK0_ORB	CLOCK1_ORB
WHU0OPSULT	NONE	02D_05M_ORB.SP3	NONE		CLOCK0_ORB	CLOCK1_ORB

**Table 3:** Methods and models used.

Items	Models/Methods
Positioning mode	static mode
PPP model	conventional PPP model using undifferenced dual-frequency code and phase ionosphere-free linear combination
Sessions	eight three-hour sessions per day (fifty-six sessions for each station from seven days for each solution type)
Signals	L1/L2 for GPS and E1/E5a for Galileo
Stochastic modeling	$\sin\left(\mathrm{elevation}\right)$
Constellation	GPS (G), Galileo (E), and GPS+Galileo (GE)
Cut-off elevation angle	7°
Interval estimation	30 s
Periods	seven days: from 152 DoY to 158 DoY of 2025
Reference frame	IGS20
Reference coordinates	daily IGS SINEX files
PCO and PCV for receiver and satellite antenna	igs20.atx
Ionospheric delay	ionosphere-free linear combination
Tropospheric delay	simple nominal with Niell mapping; estimate wet troposphere residual [54]
Solid tides, ocean loading, Shapiro effects, and phase wind-up	IERS convention 2010 [55]
Ambiguities	float
Receiver clock correction	estimated for GPS and with ISB for Galileo

## Data Availability

The data and products analyzed during the current study are publicly available in the International GNSS Service (IGS) repository, which provides open access.

## Abbreviations

The following abbreviations are used in this manuscript:

AC	Analysis Center
ACN	Analysis Center Information Notes
ADEV	Allan Deviation
AR	Ambiguity Resolution
CLK	Clock Products Format
CLAS	Centimeter-Level Augmentation Service
CNES/CLS	Centre National d’Études Spatiales / Collecte Localisation Satellites
CODE	Center for Orbit Determination in Europe
DEM	Demonstration
DoY	Day of Year
EKF	Extended Kalman Filter
FIN	Final Products
GFZ	Helmholtz Centre Potsdam GFZ—German Research Centre for Geosciences
GNSS	Global Navigation Satellite System
GPS	Global Positioning System
GRG	CNES/CLS Analysis Center
HAS	High-Accuracy Service
IERS	International Earth Rotation and Reference Systems Service
IGS	International GNSS Service
LEO	Low Earth Orbit
MGEX	Multi-GNSS Experiment
NWP	Numerical Weather Prediction
OPS	Operational Products
PHM	Passive Hydrogen Maser
PPP	Precise Point Positioning
QZSS	Quasi-Zenith Satellite System
RAP	Rapid Products
Rb	Rubidium (Atomic Clock)
RTK	Real-Time Kinematic
RTS	Real-Time Service
SP3	Standard Product 3 Format
ULT	Ultra-Rapid Products
WHU	Wuhan University
WUM	Wuhan University MGEX Analysis Center
ZTD	Zenith Tropospheric Delay
